# Critically appraised topic on adverse food reactions of companion animals (7): signalment and cutaneous manifestations of dogs and cats with adverse food reactions

**DOI:** 10.1186/s12917-019-1880-2

**Published:** 2019-05-09

**Authors:** Thierry Olivry, Ralf S. Mueller

**Affiliations:** 1 Department of Clinical Sciences, College of Veterinary Medicine, North Carolina State University, 1060 William Moore Drive, Raleigh, NC 27607 USA; 2 Medizinische Kleintierklinik, Centre for Clinical Veterinary Medicine, Ludwig Maximilian University, Veterinärstrasse 13, 80539 Munich, Germany

**Keywords:** Allergy, Canine, Cat, Clinical signs, Dermatology, Diet, Dog, Feline, Food allergy, Skin

## Abstract

**Background:**

Outside of pruritus, there is no clear consensus on the nature and prevalence of cutaneous manifestations of adverse food reactions (AFRs) in dogs and cats.

**Results:**

We searched two databases on August 7, 2018, for articles reporting detailed data on the signalment and clinical signs of at least one dog or cat with a cutaneous AFR (CAFR). We identified 233 and 407 citations from which were selected 32 articles reporting original information. Twenty-two articles included data on 825 dogs with CAFRs. The reported age of onset varied from less than one to 13 years of age; a beginning of signs by 6 or 12 months of age was noted in 22 to 38% of dogs, respectively. The female-to-male ratio also varied considerably. Four breeds (German shepherd dogs, West Highland white terriers, Labrador and golden retrievers) accounted for about 40% of affected dogs. Most dogs diagnosed with a CAFR were pruritic, most often in a generalized pattern, with the ears, feet, and abdomen also being frequently affected; the perineum was uncommonly targeted, however. Canine CAFRs presented mainly as recurrent bacterial skin infections, otitis externa and atopic dermatitis. Twelve articles reported novel information on 210 cats with this syndrome. There was no apparent breed and gender predisposition for feline CAFRs, but cats appeared to develop signs later than dogs with the same syndrome. Most cats with a CAFR were pruritic, especially on the head/face and neck, with the abdomen and ears also commonly involved. Symmetric self-induced alopecia, a head-and-neck self-traumatic dermatitis, miliary dermatitis and variants of eosinophilic diseases were the most common manifestations of feline CAFRs.

**Conclusions:**

CAFRs affect dogs and cats of any age, any breed, and both genders, with the proportion of juvenile dogs diagnosed about twice that of cats. There are no reliable breed predisposition data. Most patients are pruritic, with half the dogs having generalized pruritus and half the cats scratching their face/head or neck. Canine CAFRs most often manifest as bacterial skin infections, otitis externa or atopic dermatitis; cats with CAFRs will exhibit the expected clinical phenotypes associated with feline hypersensitivity dermatitides.

**Electronic supplementary material:**

The online version of this article (10.1186/s12917-019-1880-2) contains supplementary material, which is available to authorized users.

## Background

Adverse food reactions (AFRs) are diagnoses commonly given to dogs and cats with allergic diseases [1]. These AFRs can manifest clinically with either noncutaneous (e.g., vomiting, diarrhea) [2] or cutaneous clinical signs. While pruritus is widely accepted to be the main symptom that affects pets with a cutaneous AFR (CAFR), there is a lack of consensus on the typical signalment and cutaneous manifestations of AFRs in dogs and cats.

## Clinical scenario

You have two itchy patients: one is a three-year-old male castrated German shepherd dog with a two-year history of nonseasonal recurrent facial rubbing and pedal licking. On physical examination, you notice erythema on the groin, the palmar metacarpi, and the concave pinnae. The second is a two-year-old female spayed domestic shorthaired cat with a six-month history of severe and nearly continuous head-and-neck scratching that leads to the development of large facial excoriations. You wonder if the histories, signalment and clinical signs of your two patients would be compatible with a CAFR.

## Structured question

*What are the typical signalment and cutaneous manifestations of AFRs in dogs and cats?*

## Search strategy

We searched the Web of Science Core Collection and CAB Abstract databases on August 7, 2018 with the following string: ((dog or dogs or canine) or (cat or cats or feline)) and (food* or diet*) and (allerg* or reaction*) and (prurit* or cutan* or skin) not (human* or adult* or child*). This search was restricted to the January 1980 to July 2018 timeframe, and we did not set any publication language limits. The bibliography of each selected article was subsequently screened for additional relevant papers. Because of the need for detailed information, we did not search conference proceedings, as we deemed abstracts to be too succinct to allow for the extraction of quality and pertinent data. We limited our search to articles reporting the signalment and clinical signs of at least one dog or cat with a CAFR. Finally, we did not consider review papers because of our need for original information.

## Identified evidence

Our search identified 233 and 407 citations in the Web of Science and CAB abstracts, respectively. Among these citations, we found a majority of review papers, but we located 32 articles reporting novel, relevant and usable data. Importantly, 13 of these 32 articles were found in only one of the two databases searched, thereby highlighting the need to query multiple sources to maximize evidence identification; we added only one additional paper found in the bibliography of another. Altogether, reports included pets with CAFRs seen all over the world: cases were from Europe (16 articles), North (8) and South (2) America, Australia (2), Africa (1), Asia (1); two articles were global surveys (Additional file 1: Table S1 and Additional file 2: Table S2).

## Evaluation of evidence

Twenty articles reported information on dogs with CAFRs [3–22] while there were ten papers describing such cats [23–32]; two included patients of both species. [33, 34].

In this paper, and in an evaluation scheme similar to that of our recent review [2], we rated the quality of CAFR diagnosis as “*strong*” if the study was prospective and the diagnosis confirmed by a positive challenge that followed an elimination diet. We qualified the diagnosis strength as “*moderate*” if the study was retrospective in nature but included both restriction and provocation phases; otherwise, we assessed the diagnosis quality as “*weak*”.

### Canine cutaneous adverse food reactions

Altogether, we reviewed data on 825 dogs with CAFR (mean: 38 per paper; range: 1 to 172). In these dogs, the evidence for this diagnosis was assessed as strong in 284 (34%), moderate in 339 (41%) and weak in 203 (25%) (Additional file 1: Table S1).

**Fig. 1:**
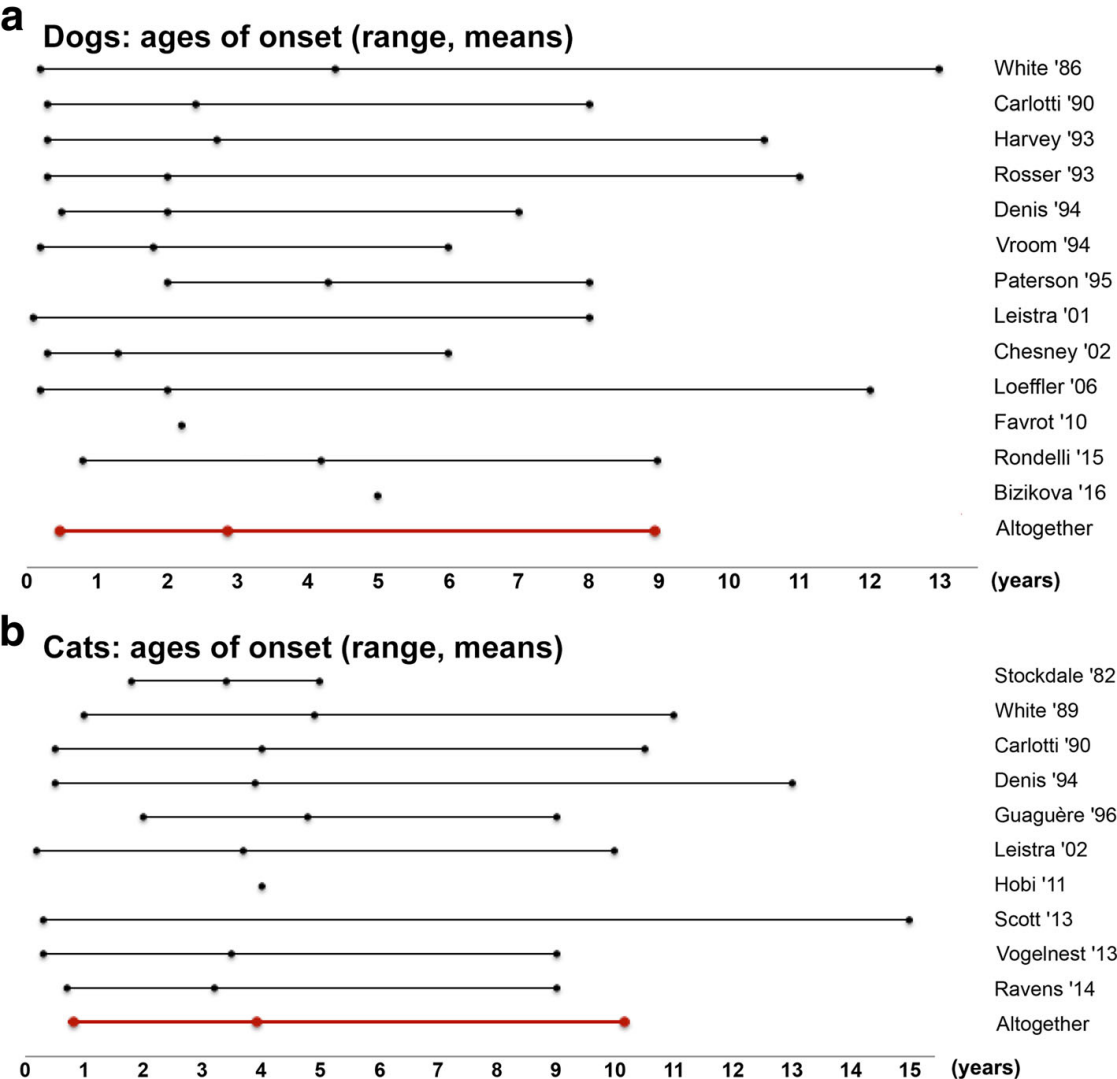
Ages of onset of cutaneous adverse food reactions in dogs and cats. **a** Ages of onset of CAFRs in dogs and, **b** in cats. We only included studies reporting information on more than one animal and from which relevant data was usable. The lines represent the range and the dot inside each line depicts the means stated in that study. The red lines indicate the average of study means, as well as the minimal and maximal values of the mean ranges

The reported age of onset of canine CAFR varied greatly, from less than one to 13 years of age; pooled together, the mean age of onset was 2.9 years (Additional file 1: Table S1; Fig. 1a). An onset of signs at six months of age or before was noted in 40/182 dogs (22%) in ten articles [3, 4, 6, 10, 15, 18, 19, 22, 34]. Similarly, an onset of signs by one year of age was described in 217/574 dogs (38%) in 14 articles [3–6, 8–10, 12, 15–19, 22].

**Fig. 2:**
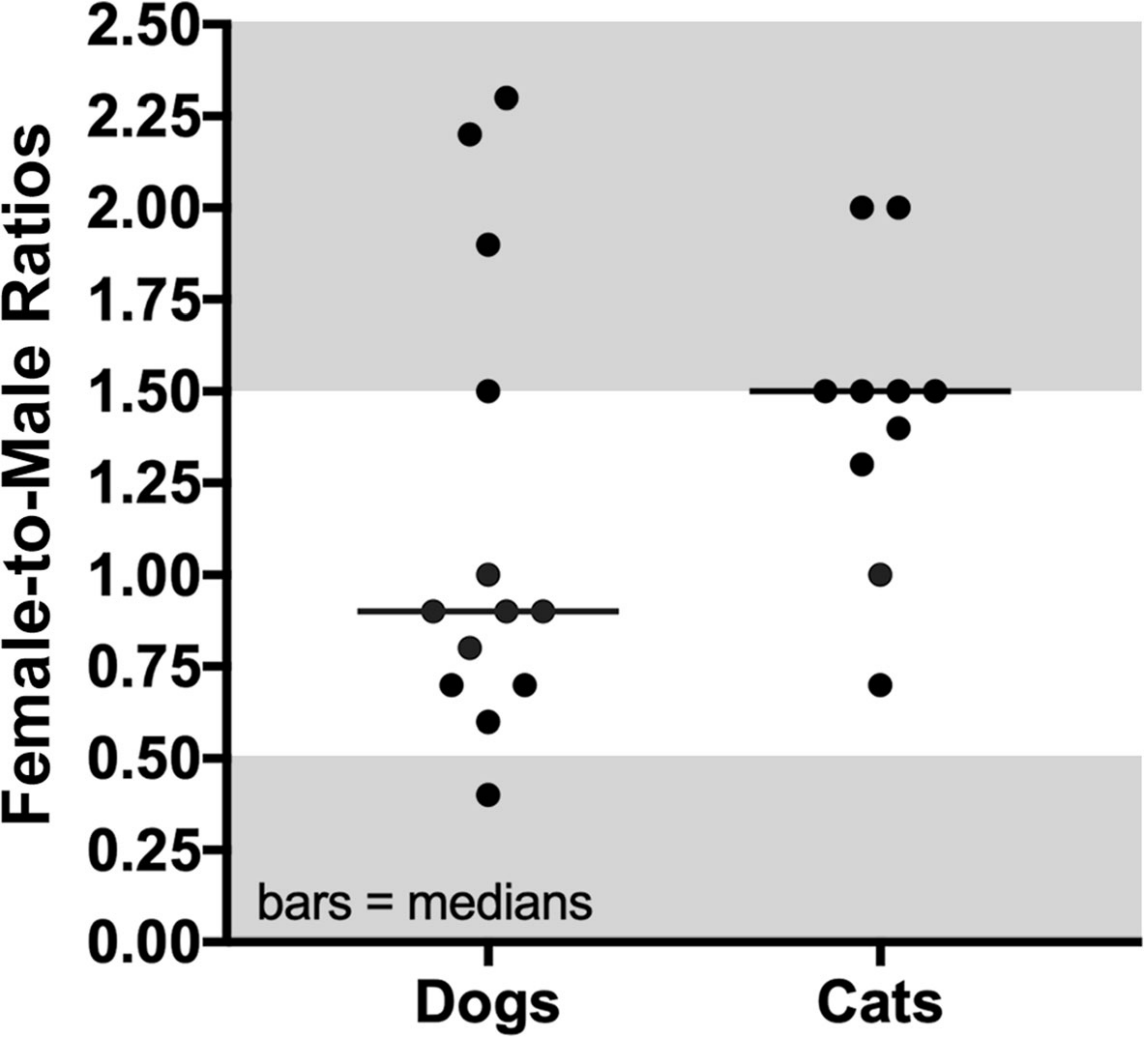
Female:male ratios in dogs and cats with cutaneous adverse food reactions. Each dot represents a study from which the relevant data were extracted. The bars depict the medians. Studies outside the shaded (grey) areas are those with an over-representation of females or males

In 13 studies reporting information on more than one dog (483 patients in total) [3–7, 9, 10, 14, 16, 17, 20, 21, 33], CAFRs affected both male and female dogs in a proportion that varied greatly between reports: while the median female-to-male ratio was 0.9, some studies reported a higher proportion of either males (a ratio of 0.4) or females (ratios of 1.5 to 2.3 – Additional file 1: Table S1; Fig. 2).

In the Additional file 1: Table S1, we extracted the breeds representing at least 10% of the dogs (with a minimum of three) included in each case series. Altogether, there were nine reports with 57/432 dogs (13%) being German shepherd dogs [4, 8, 11–14, 16, 33, 34], six articles with 40/209 dogs (19%) being Labrador or golden retrievers [4–6, 8, 10, 13] and five papers [3, 6–8, 13] describing 18/164 dogs (11%) as West Highland white terriers; these observations suggest the persistence of CAFR diagnosis in these breeds over time and geographical areas. A comparison with a reference canine population was only done in nine studies, however [4, 5, 9, 10, 12, 13, 16, 33, 34]. In four of these articles [4, 9, 16, 33], a significant association between a breed and the diagnosis of CAFR was not found, while in the five others, some breeds appeared predisposed to this syndrome when compared to the then local canine population (Additional file 1: Table S1). While we did not identify a breed consistently associated with CAFR, both Labrador retrievers [5, 10, 34] and West Highland white terriers [5, 12, 13] were predisposed breeds in three reports each.

Pruritus was the dominant symptom reported in 16/17 articles (94%). Overall, and excluding a single dog affected with a nonpruritic acute eosinophilic dermatitis with edema (i.e., Wells’ syndrome) [18], 13/16 articles (81%) reported more than 90% of the included dogs as being pruritic. The pruritus was characterized as glucocorticoid-responsive in two studies [13, 17] (Additional file 1: Table S1).

The main body locations in which pruritus was present varied between reports (Additional file 1: Table S1): studies in which more than half of the dogs exhibited a specific pattern of pruritus described it as generalized [3, 19, 22, 33] or affecting the ears [4, 5, 16], feet [5, 15] or ventrum [5, 17]. In contrary to commonly-held beliefs, perineal pruritus, when reported, affected only a minority (4 to 25%) of dogs with CAFRs [4, 5, 9, 16, 20, 34].

There was much heterogeneity in the reporting of cutaneous manifestations of AFRs in the dog, with some studies describing individual skin lesions (e.g., erythema, alopecia …) and others mentioning specific diagnoses (e.g., atopic dermatitis, urticaria …) or syndromes without further details (e.g., otitis externa, recurrent pyoderma...) (Additional file 1: Table S1).

Excluding studies with single lesion descriptions, the most common manifestations of canine CAFRs (reported in more than one paper) were various presentations of often-recurrent or chronic (presumed staphylococcal) pyoderma (i.e., bacterial skin infections; ten reports with between 11 and 70% of dogs affected [3–5, 10, 13, 16, 17, 20, 33, 34]), otitis externa (nine studies: 3 to 69% of dogs [3–5, 10, 13, 14, 17, 33, 34]), atopic dermatitis (AD, nine reports; 13 to 100% of dogs [3, 7, 8, 10, 11, 13, 17, 33, 34]) and pyotraumatic dermatitis (four studies; 1 to 9% of dogs [5, 17, 33, 34]) (Additional file 1: Table S1). It is needless to add that multiple manifestations of CAFRs often coexisted in the same patient (Additional file 1: Table S1).

*Malassezia* dermatitis, urticaria and perianal fistulae/furunculosis were reported as manifestations of CAFRs in a surprisingly small number of dogs and reports (93 dogs/two articles [13, 17] nine dogs/four articles [17, 19, 22, 33] and four dogs/two articles [16, 33] respectively; Additional file 1: Table S1).

### Feline cutaneous adverse food reactions

In total, we extracted relevant information from 210 CAFR-affected cats (median: 14 cats in each article; range: 1 to 61). In these cats, the evidence for a diagnosis of CAFR was rated as strong, moderate or weak in 22 (10% of cats), 175 (83%) and 13 (6%), respectively (Additional file 2: Table S2).

As in dogs with this syndrome, the age of onset of cutaneous signs in cats with CAFRs varied greatly between and within reports (Additional file 2: Table S2; Fig. 1b). For example, signs were described as occurring as early as 4 months and as late as 15 years of age in a single study [30]. Altogether, the mean age of sign onset of feline CAFRs was 3.9 years. A development of cutaneous signs by 6 months of age was reported in 6/70 cats (9%) in seven articles [23–25, 27, 31, 32, 34], while that by 1 year of age was described in 16/70 cats (23%) in the same articles.

The median female-to-male ratio of cats diagnosed with a CAFR was 1.5, with only two studies having females seeming over-represented (a ratio of 2.0; Additional file 2: Table S2; Fig. 2) [28, 29].

Outside of the domestic shorthaired cat that was ubiquitously listed, Persian, Siamese, and Burmese cats represented 10 (5%), 8 (4%) and 4 (2%) of all felines with CAFR, respectively. Only three articles had compared affected breeds with those of the then local population, and there were no breeds appearing predisposed across reports (Additional file 2: Table S2) [30, 31, 34].

Nearly all cats with CAFRs exhibited manifestations of pruritus [23–25, 27–34]. A single cat was described as having nonpruritic cervical nodules [26] (Additional file 2: Table S2).

The pruritus was described as generalized only in a small percentage (5 to 12%) of cats in two reports [28, 31]. Tallying all cases together, the face/head was pruritic in half of the cats reported (111/210 [53%]) [23–25, 27–34]. Other areas commonly found to be pruritic were the ears (18 to 54% of cats published in seven articles [23, 24, 27, 29–31, 34]), the ventrum (25 to 66% of cats in six studies [27–29, 31, 32, 34]) and the feet (6 to 33% of cats in six reports [27, 29–32, 34]. As in dogs, the perineum was not an area frequently pruritic in cats with a CAFR, however (10 to 15% of cats in three articles [28, 29, 34]).

There was much heterogeneity in the reporting of skin lesions of feline CAFRs, as in dogs with this syndrome. In studies with more than one cat, the most commonly described clinical presentations were a presumed self-induced, often-symmetric alopecia (40 to 100% of cats in nine articles [23, 24, 27, 29–34]), a head-and-neck erosive/ulcerative/crusted dermatitis (30 to 65% of cats in three such studies [27, 29, 33]), the papulo-crusted miliary dermatitis (21 to 40% of cats in seven articles [24, 27, 29–33]) or variants of eosinophilic diseases (6 to 23% of cats in five reports [24, 27, 29, 30, 33]). In the most recent studies, cats were diagnosed as having a concurrent non-flea-associated hypersensitivity/AD in 19 to 100% of included cases [30–32].

## Limitations

Several factors could limit the generalization of the findings to the population of dogs and cats with CAFRs. For example, the studies included spanned more than 30 years, and the methods, strictness, precision, and nomenclature of diagnoses evolved both over time and geographical areas, thus leading to some possible confusion. Furthermore, some of the manifestations of CAFRs (e.g., *Malassezia* dermatitis) were not recognized until the late 1980’s. The lack of comparison of signalment data with the then local companion animal population—and the relatively low number of affected individuals of rarer breeds in some studies—prevents an accurate assessment of age, sex and breed predispositions. The lack of reporting of long-known manifestations of CAFRs might lead to the erroneous perception that some diseases (e.g., food-induced urticaria) are rarer than in reality. Finally, some studies were limited to dogs with AD, and this could be a source of publication bias favoring the over-diagnosis of food-induced AD (FIAD). Of importance is that we could not separate cases with a* bona fide* FIAD from those with a CAFR with a concurrent yet not food-related AD.

## Conclusion and implication for practitioners

In summary, CAFRs affect dogs and cats of nearly all ages and both genders, with the onset of clinical signs likely occurring later in cats than in dogs. Almost 40% of dogs develop cutaneous manifestations of AFR by one year of age, while this happens in about half that in cats. There are four canine breeds (German shepherd dogs, Labrador, and golden retrievers and West Highland white terriers) that account for over four of ten dogs with CAFR, but there is no reliable evidence of unique canine and feline breed predispositions to develop CAFRs.

Most dogs and cats with CAFRs appear to be pruritic, making this symptom a sensitive—albeit nonspecific—sign for such syndrome. While dogs with CAFR are affected more often with a generalized pruritus than cats with the same diagnosis, cats have more pruritic faces, heads and necks than dogs; other commonly pruritic areas in dogs and cats with CAFRs are the ears, ventrum, and feet. In contrary to current beliefs, the perineum is not usually the target of pruritic manifestations in either species.

In dogs, the most often reported cutaneous manifestations of an AFR are recurrent bacterial and yeast skin infections, otitis externa and AD, which can all coexist in the same patient. In cats, CAFRs manifest as the expected syndromes associated with hypersensitivities, such as a usually-symmetric self-induced alopecia, a head (face)-and-neck self-traumatic dermatitis, the miliary dermatitis and variants of eosinophilic diseases.

## Additional files

Additional file 1: **Table S1.** Summary of articles reporting dogs with cutaneous adverse food reactions. (XLSX 17 kb)

Additional file 2: **Table S2.** Summary of articles reporting cats with cutaneous adverse food reactions. (XLSX 16 kb)

## Abbreviations

AD: Atopic dermatitis
AFR(s): Adverse food reaction (s)
CAFR(s): Cutaneous adverse food reaction(s)
CAT: Critically-appraised topic
FIAD: Food-induced AD
